# Effect of symmetrical restoration for the migration of uncemented total hip arthroplasty: a randomized RSA study with 75 patients and 5-year follow-up

**DOI:** 10.1186/s13018-020-01736-0

**Published:** 2020-06-17

**Authors:** Sverrir Kiernan, Mats Geijer, Martin Sundberg, Gunnar Flivik

**Affiliations:** 1 Department of Orthopedics, Skåne University Hospital, Clinical Sciences, Lund University, Lund, Sweden; 2 Department of Radiology, Institute of Clinical Sciences, Sahlgrenska Academy, University of Gothenburg, Gothenburg, Sweden; 3 Department of Radiology, Region Västra Götaland, Sahlgrenska University Hospital, Gothenburg, Sweden; 4 Department of Clinical Sciences, Lund University, Lund, Sweden

**Keywords:** THR, THA, RSA, Radiostereometry, Anatomical restoration, 3D-CT

## Abstract

**Background:**

Inferior placement of a femoral stem is predictive for early loosening and failure, but does restoration of the original hip anatomy benefit the function and survival of a total hip replacement?

**Methods:**

Seventy-five patients with primary unilateral hip osteoarthritis operated with an uncemented anatomical stem were randomized for either standard or modular stems. We used 50 ABG II stems with modular necks and 25 standard stems (control group). We measured the symmetry in hip anatomy between healthy and operated side. The anatomical restoration variables were anteversion, global offset, and femoral offset/acetabular offset (FO/AO) quota. We performed measurements using a CT-based 3D templating and measuring software. Migratory behavior of the stems was then measured postoperatively with repeated radiostereometry (RSA) examinations over 5 years.

**Results:**

Both stem types showed an early (within 3 months) good stabilization after an initial slight rotation into retroversion and subsidence. There were no significant differences in RSA migration between modular and standard stems. Postoperative anteversion and FO/AO quota had no impact on stem migration. The standard stem tended to result in insufficient global offset (GO), whereas the modular stem did not.

**Conclusions:**

The modular stem gave good symmetrical anatomical restoration and, like the standard version, a benign migratory behavior. Anteversion, GO, and FO/AO quota had no significant impact on stem migration. It therefore seems to be of no importance whether we choose a modular or a standard stem with regard to postoperative stem migration for this stem type. We overestimated the effect anatomical parameters have on stem movement; hence, we believe the study to be underpowered.

**Trial registration:**

ClinicalTrials.gov identifier: NCT01512550. Registered 19 January 2012—retrospectively registered,

## Background

Restoring hip anatomy is important for function [1–4], but more studies are needed to determine the importance of restoration for survival of the total hip replacement (THR) [1]. An endoprosthesis can better withstand various load factors and function better if positioned according to the original anatomy. Too small femoral offset (FO) is associated with increased acetabular polyethylene wear [5] and improving lever arm biomechanics by increasing FO reduces the load transferred to the cup [6].

Too small (< 10°) anteversion appears harmful to the long-term outcome for cemented femoral stems [7]. Leg-length-discrepancy (LLD) can result in biomechanical changes in hip joint load both on the long and the short side, which may cause problems in the long term [8]. The size of clinically significant LLD is however unclear [9]. 2D templating software systems have been developed to facilitate anatomical restoration [10] and there is even an increasing interest to advance from 2D projections to more accurate 3D measurements [11, 12]. Computer-assisted surgery (CAS) [13] can also be used to facilitate the placement of a prosthesis. The use of modular necks has been suggested to facilitate anatomic restoration [14], but not much is known about other biomechanical effects of increased modularity [15]. Today, there have been no studies reporting the effect of stem modularity on the migratory behavior of the stem. We tested the hypothesis that restoration of the hip anatomy benefits the migration behavior of the stem and that a modular stem system can be beneficial to reach the planned positioning of the implant, reducing the risk of unfavorable biomechanical strain.

## Methods

In a randomized prospective cohort study, we analyzed stem migration with successive RSA examinations during 5 years follow-up. Our study group consisted of 75 patients (48 males, 27 females) with primary osteoarthritis (OA) of the hip undergoing THA between October 2009 and September 2011. Inclusion criteria were patients less than 75 years of age with primary unilateral OA of the hip. We only considered patients with bone quality and morphology of the proximal femur suitable for an uncemented stem, i.e., type A and some type B femurs according to the Dorr classification [16]. Patients who were capable of understanding the conditions of the study with CT-scans and RSA at follow-up and who were willing to participate for the duration of the prescribed follow-up were asked to enroll and had to give their written informed consent to participation. The mean age at the time of operation was 59 (34–80) years and mean BMI was 29 (20–36). Seventy-four out of 149 initial patients did not fulfill the inclusion criteria due to a bone quality and morphology according to our criteria obviously unsuitable for an uncemented stem or due to the fact that a standard stem was inadequate for anatomical restoration as offset of the stem increases with size resulting in incompatibility between offset and size fit (Consort flow Diagram).

**Fig. 1:**
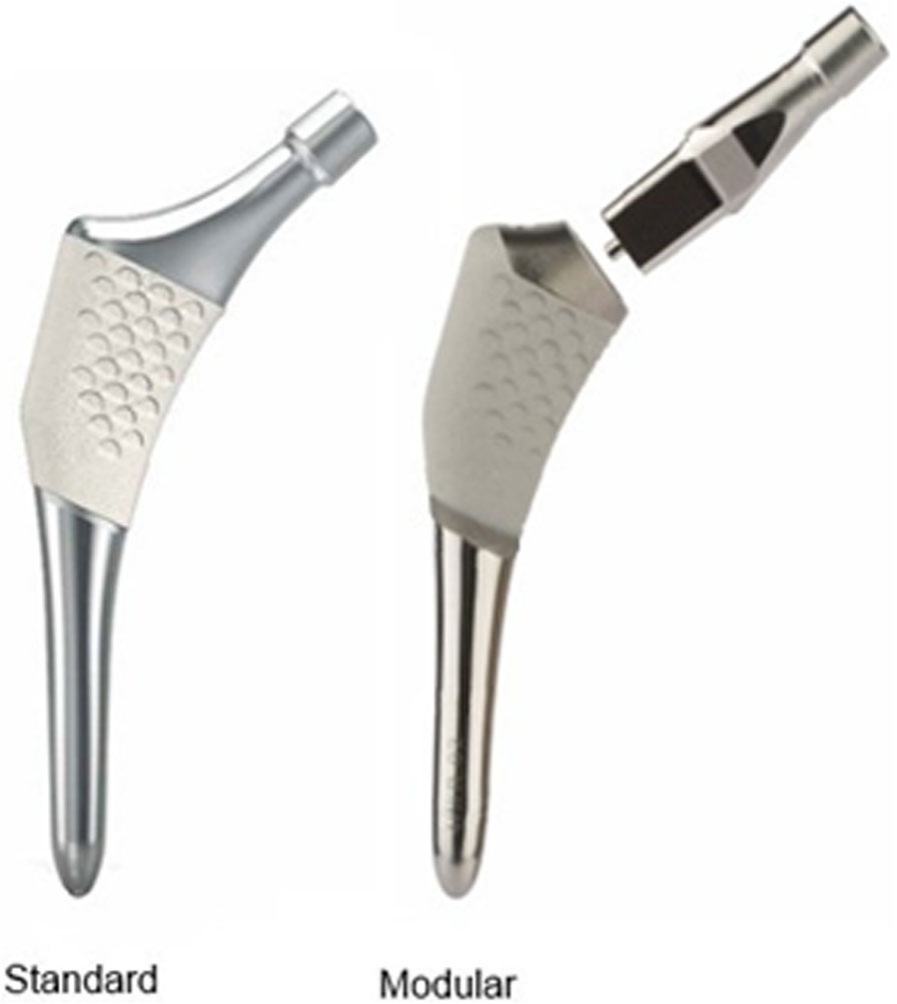
ABG II system: standard and modular stems

We prepared 75 envelopes randomized for 50 modular stems (ABG II modular® hip and Trident® Acetabular system (Stryker Orthopaedics, Mahwah, New Jersey, USA)) and 25 standard stems (ABG II monolithical® and Trident® Acetabular system (Stryker Orthopaedics, Mahwah, New Jersey, USA)) (Fig. 1). The latter was our control group. The ABG II monolithical® Hip Stem is an anatomical stem intended for cementless, press-fit application and is designed for the best proximal anatomical fit. The proximal region of the stem is coated with PureFix® HA. The standard system includes left and right stems with 8 body sizes ranging from size 1 to size 8 in which offset increases with size. The modular version has the same stem body but comes with enhanced alignment abilities, to choose the most suitable modular neck for extramedullary anatomic fitting.

### Computed tomography

We performed two separate CT scans pre- and postoperatively using a low-dose technique, with an effective radiation dose exposed to the patient equivalent to that of conventional radiography [17]. CT was performed directed over the pelvis and hips, and a separate scan covering the knees. The pelvic scan was planned from slightly cranial to the superior anterior iliac spine to about 3 cm below the lesser trochanter. The knee scan aimed at inclusion of the femoral condyles and a few centimeters of the proximal tibia. We performed the CT on a multi-detector helical Brilliance 64 CT scanner (Philips, Eindhoven, The Netherlands). We used low-dose settings for the preoperative study and a medium-dose setting to compensate for the implanted prosthesis for the postoperative study. CT dose index by volume (CTDIvol) was set as 4.8 for the preoperative hip study and 4.2 for the preoperative knee study, whereas CTDIvol was 16.4 for the postoperative hip study to compensate for the hip arthroplasty but the knee dose was unchanged.

### Preoperative templating

The surgeon did a preoperative 2D templating based on conventional calibrated radiographs. The X-rays were produced in a standardized manner where we centered the anteroposterior view of the pelvis on the symphysis pubis, with toes touching to control femoral rotation. The templating was done with the contralateral healthy hip anatomy as reference but also in part based on the measurements previously done on preoperative 3D-CT measurements. This gave the surgeons the means to choose the correct stem size, and in the case of the modular stems, with enhanced alignment abilities, to choose the most suitable modular neck for extramedullary anatomic fitting.

For templating, we used Sectra IDS7 PACS Orthopaedic Package^TM^ (Sectra AB, Linköping, Sweden).

### Surgical procedure

Two experienced hip surgeons performed the operations through a posterolateral approach. Before stem implantation, we marked the proximal femur with 9 to 10 tantalum markers (diameter 0.8 mm), with 3 to 4 in the lesser trochanter and 5 to 6 in the greater trochanteric area.

**Fig. 2:**
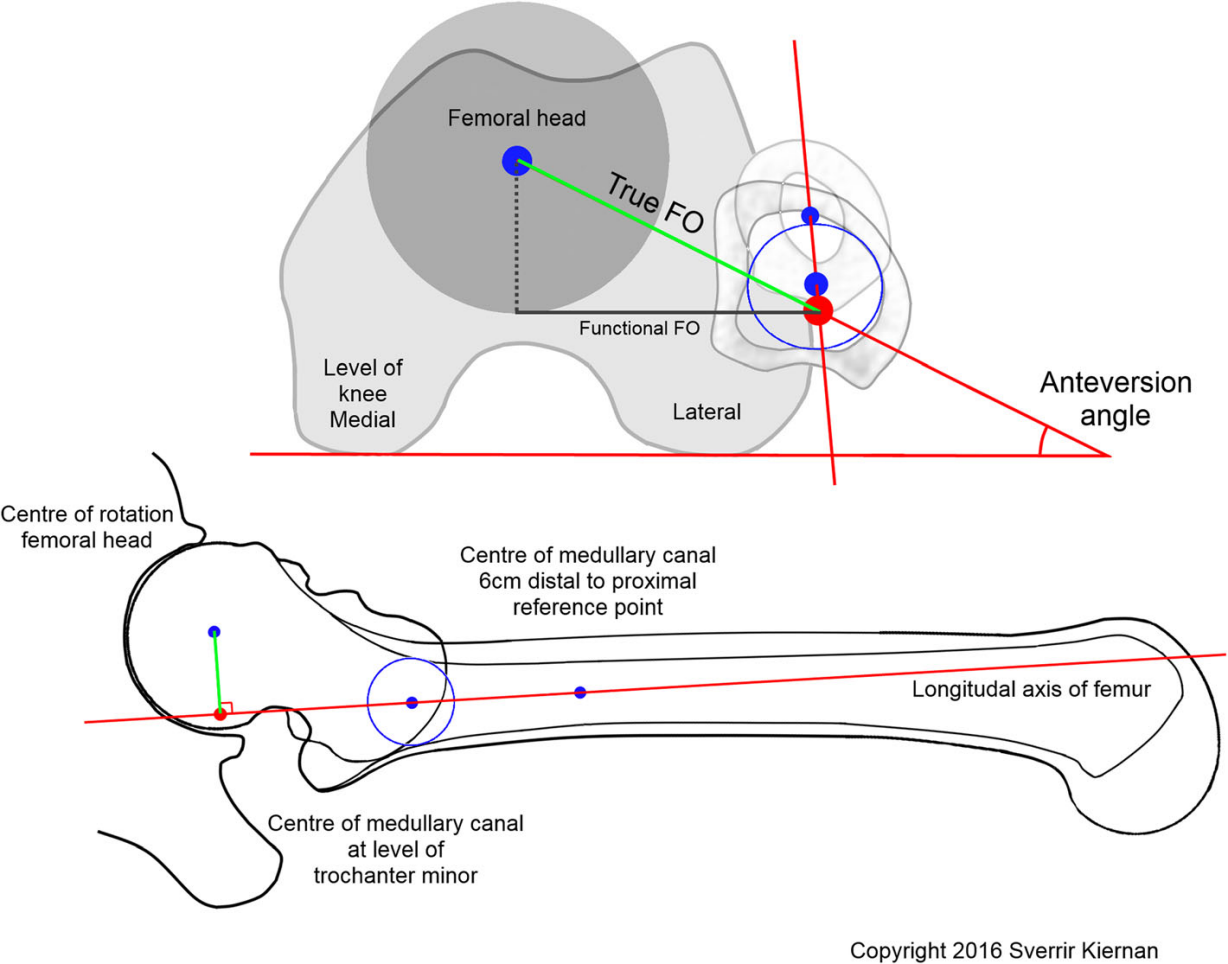
Reference points for 3D-CT measurement of anteversion. A proximal reference point in the center of the medullary canal at the lower level of trochanter minor and a second 3 cm more distal reference point in the center of the medullary canal formed the longitudinal axis of the femur. The perpendicular line (femoral offset) runs from the longitudinal axis of the femur to the center of rotation. The hip anteversion is the angle between this perpendicular line in relation to the posterior femoral condylar line

**Fig. 3:**
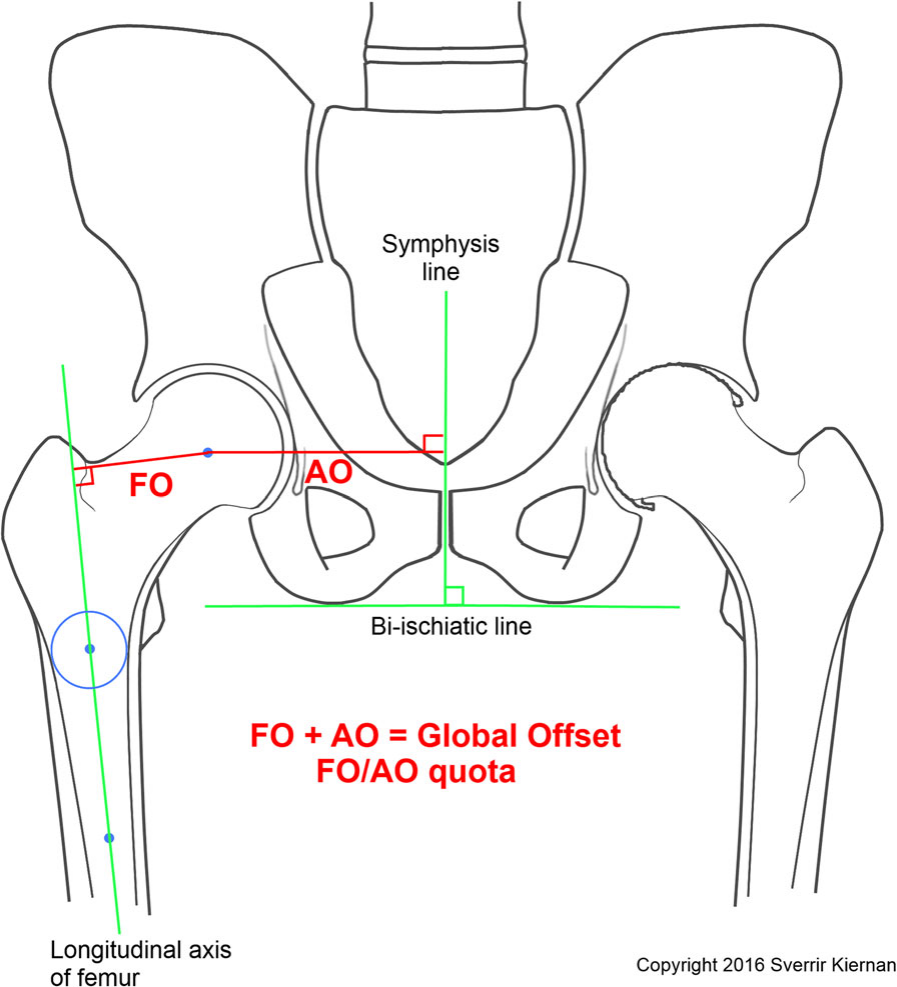
Femoral offset (FO) is the distance between the longitudinal axis of the femur to the center of rotation. Acetabular offset (AO) is the distance between the center of rotation to the symphysis line. Global offset is the FO plus the AO

The ABG II modular stem is anatomical and cementless and therefore orients itself into best proximal fit. However, well in place, the surgeons had the option to use one of three neck versions (retroverted, standard, and anteverted) in order to mimic the contralateral healthy hip with preoperatively measured (3D-CT) anteversion (Fig. 2). Anatomical restoration of global offset was attempted to mimic the global offset of the contralateral healthy hip measured during the preoperative 2D templating procedure (Fig. 3).

### 3D-CT measurements

An independent observer made all measurements on pre- and postoperative 3D-CT without knowledge of previous measurements and had no knowledge or involvement in preoperative 2D templating or the patients’ management. The pre- and postoperative 3D-CT examinations were assessed for lever arms and rotatory positions of the stems, using a CT-based 3D templating software (Ortoma Plan^TM^, Gothenburg, Sweden). This software gives validated highly accurate measurements for these variables. The interrater reliability results for the 3D-CT measures were generally near perfect for all our variables with high interclass correlation coefficients (0.887 to 0.974) and narrow confidence intervals for the two raters. We will report these results in a separate paper. The variables for anatomical restoration were the symmetry of anteversion, global offset, and the FO/AO quota in relation to the healthy hip.

### Radiostereometric analysis

RSA was carried out using a uniplanar technique with the patient supine [18]. Two X-ray sources were fixed, mounted to the ceiling. We used a type-41 calibration cage (Tilly Medical, Lund, Sweden) and the MBRSA 4.0 computer software version 4.0 (Leiden, Holland). We used model-based RSA (MBRSA, Leiden, Netherland) with an elementary geometry shape (EGS) to add two fictive markers to the stem, one at the tip of the stem and one in the center of rotation in the head of the prosthesis. The reference examination was performed on the first postoperative day and served as the reference for all further analyses. Follow-up examinations were carried out after 2 weeks, 3 months, and at 1, 2, and 5 years, with a time tolerance of 5% at each interval. We set the cut-off level for the exclusion of patients or of specific examinations at a condition number of 150 (An expression for how well spread the tantalum markers are in the segment. Better spread will result in lower CN and more reliable RSA results). For the mean error of rigid body fitting (an expression for marker stability), the cut-off level was set at 0.3 [19].

RSA values were expressed as migration (rotation and translation) about/along the 3 axes in an orthogonal coordinate system (6° of freedom) and referred to as transverse (*x*-axis), longitudinal (*y*-axis), and sagittal (*z*-axis). We considered distal translation (subsidence) and longitudinal rotation (both in/about the *y*-axis) as primary effect variables for how the stem migrates. We had 75 double examinations for precision assessment of our RSA measurements (Table 1).

**Table 1:** Precision of radiostereometric analysis for assessment of stem migration

**Axis**	Translation (mm)*	Rotation (°)*
Transverse (*x*)	0.23	0.46
Longitudinal (*y*)	0.18	1.14
Sagittal (*z*)	0.30	0.24

*Precision of measurements based on 75 double investigations. Given number represents the smallest migration value that is considered significant and is based on 2 standard deviations of the error obtained. This, hence, represents the 95% confidence limit

### Statistical analysis

**Fig. 4:**
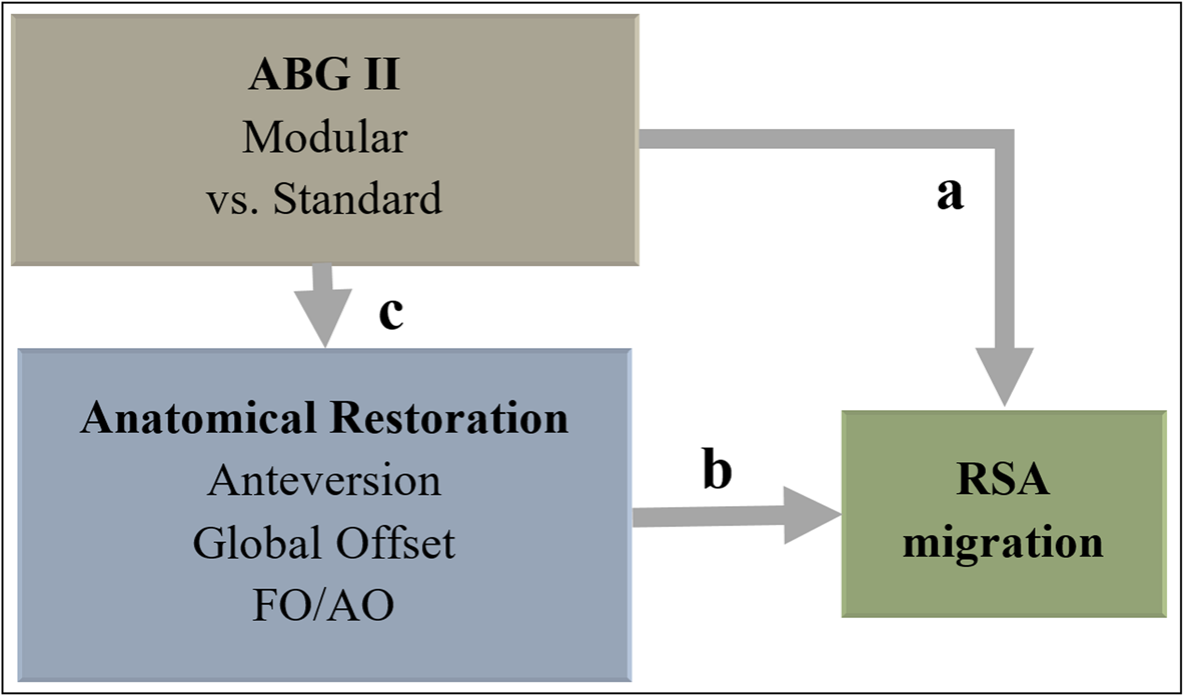
Overview of statistical analysis and variables

We used a variance adjusted mixed model to analyze migrating behavior in relation to stem type (Fig. 4a) where we treated patient ID as a random effect. We used logistic regression to analyze postoperative anatomical symmetry. We were interested in whether better symmetry (where the non-operated leg was a reference) in anteversion, global offset, and FO/AO quota were significant factors to influence postoperative stem migration (Fig. 4b). When evaluating the impact of individual anatomical discrepancies on the probability of becoming at risk for increased postoperative stem migration, we chose to classify anteversion symmetry within the range of – 2.5° to + 2.5° discrepancy between hip sides. Likewise, we set the range for GO symmetry to – 2.5 to + 2.5 mm between sides. We used Fisher’s exact test to evaluate the difference in anatomical restoration regarding stem type and examined distribution histograms for precision estimates (Fig. 4c). We conducted all calculations in STATA s (IC v12 and v13).

## Results

### Radiostereometric analysis

The mean migration rates for all stems after each follow-up period are summarized in Table 2 and further divided into subgroups of stem types.

**Table 2:** Results of RSA

	**Mean stem migration (Stdev) in relation to direct postoperative reference examination**						
	**Early migration**		**Late migration**				
	**2 weeks**	**3 months**	***p*value**^**£**^	**1 year**	**2 years**	**5 years**	***p*value**^**$**^
**Rotation (°)**							
***X*-axis**							
All stems	0.15 (0.52)	0.15 (0.65)	0.13	0.09 (0.67)	0.17 (0.61)	0.27 (0.79)	**0.01**
Modular stems	0.12 (0.52)	0.11 (0.65)	0.54	0.01 (0.67)	0.12 (0.61)	0.16 (0.79)	0.18
Standard stems	0.21 (0.49)	0.24 (0.72)		0.27 (0.72)	0.28 (0.66)	0.51 (0.68)	
***Y*-axis**							
All stems	0.66 (1.27)	1.03 (1.51)	**< 0.001**	1.05 (1.41)	1.23 (1.60)	1.47 (1.70)	**< 0.001**
Modular stems	0.61 (1.27)	1.07 (1.51)	0.35	1.11 (1.41)	1.32 (1.60)	1.56 (1.70)	0.93
Standard stems	0.76 (1.49)	0.95 (1.67)		0.92 (1.61)	1.03 (1.97)	1.25 (2.02)	
***Z*-axis**							
All stems	− 0.56 (0.57)	− 0.69 (0.68)	**< 0.001**	− 0.70 (0.71)	− 0.75 (0.77)	− 0.82 (0.77)	**< 0.001**
Modular stems	− 0.55 (0.57)	− 0.69 (0.68)	0.74	− 0.69 (0.71)	− 0.76 (0.77)	− 0.81 (0.77)	0.62
Standard stems	− 0.60 (0.70)	− 0.69 (0.82)		− 0.72 (0.83)	− 0.74 (0.95)	− 0.84 (0.89)	
**Translation (mm)**							
***X*-axis**							
All stems	0.16 (0.25)	0.18 (0.26)	**< 0.001**	0.18 (0.27)	0.20 (0.29)	0.23 (0.30)	**0.001**
Modular stems	0.14 (0.25)	0.18 (0.26)	0.50	0.16 (0.27)	0.19 (0.29)	0.21 (0.30)	0.15
Standard stems	0.21 (0.29)	0.19 (0.32)		0.22 (0.33)	0.21 (0.39)	0.28 (0.33)	
***Y*-axis**							
All stems	− 0.76 (0.83)	− 1.00 (1.10)	**< 0.001**	− 1.00 (1.12)	− 0.89 (1.21)	− 0.92 (1.11)	0.09
Modular stems	− 0.70 (0.83)	− 0.88 (1.10)	0.17	− 0.88 (1.12)	− 0.84 (1.21)	− 0.86 (1.11)	0.77
Standard stems	− 0.90 (0.89)	− 1.25 (1.21)		− 1.25 (1.22)	− 1.01 (1.49)	− 1.05 (1.07)	
***Z*-axis**							
All stems	0.01 (0.26)	0.03 (0.34)	0.22	0.06 (0.42)	0.02 (0.40)	0.01 (0.44)	0.66
Modular stems	0.00 (0.26)	− 0.02(0.34)	**0.02**	− 0.03 (0.42)	− 0.04 (0.40)	− 0.09 (0.44)	**0.03**
Standard stems	0.03 (0.23)	0.14 (0.43)		0.25 (0.51)	0.14 (0.53)	0.23 (0.48)	

^£^*p* values for estimates of changes before 3 months representing the period when the stem settles in place

^$^*p* values for estimates of changes from 3 months after surgery during which osseous integration and stabilization should have occurred

**Fig. 5:**
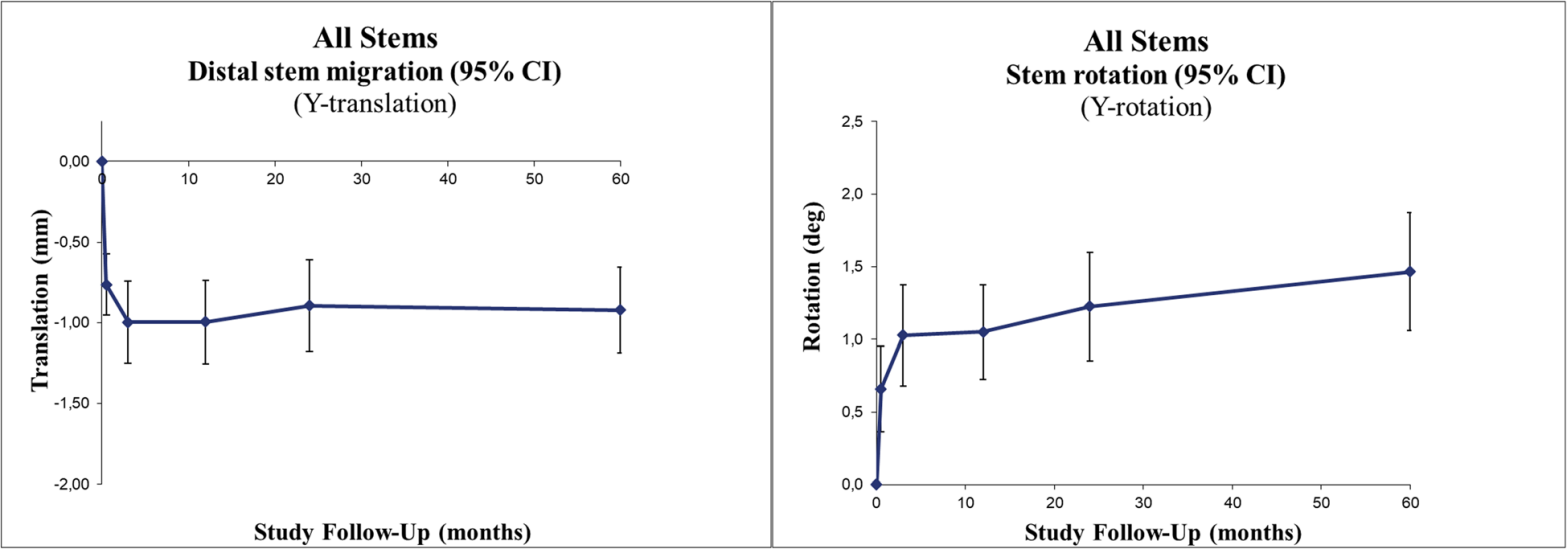
Line charts with 95% confidence intervals

The whole group showed a statistically significant mean early stem subsidence of 1.00 mm and average stem retroversion by 1.03° within the first 3 postoperative months (*p* < .0001 and *p* < .0001, respectively). After that, until the 5-year follow-up, the stems rotated slightly further to an average of 1.47° (*p* < .0001), while no more subsidence occurred after 3 months (*p* = 0.09) (Fig. 5, Table 2).

### Migrating behavior

#### ABG II modular vs. standard

**Fig. 6:**
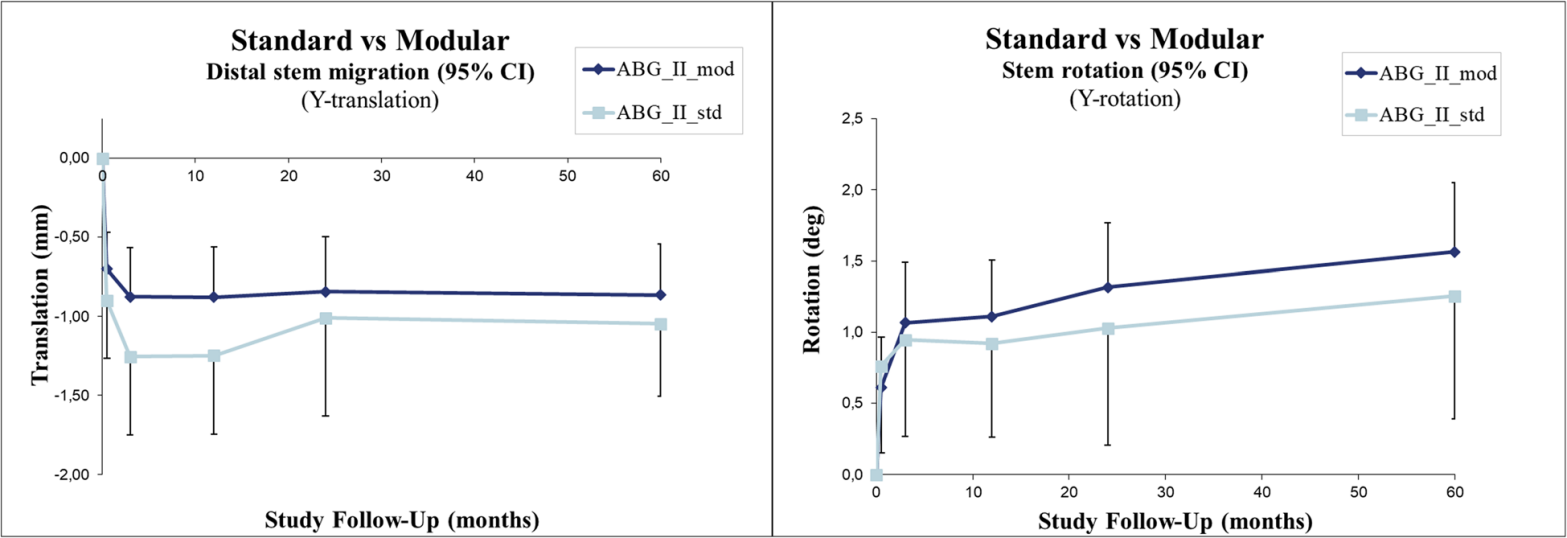
Line charts with 95% confidence intervals

Comparing the modular and standard designs, we found no difference regarding neither retroversion nor subsidence (Fig. 6, Table 2).

#### Postoperative anatomical symmetry

Postoperative stem anteversion and FO/AO quota had no impact on late postoperative stem migration.

We found no differences in postoperative stem migration related to how well hip symmetry was restored with regard to anteversion and GO.

### Stem type vs. symmetry

When comparing different stem types, there was no difference regarding symmetrical anteversion restoration (*p* = 0.20) nor symmetrical GO restoration (*p* = 0.32). However, compared to the modular stem, the standard stem had a tendency towards a lower GO on the operated side compared to the contralateral side (*p* = 0.00).

## Discussion

The results indicate an early stabilization of both stem types after an initial rotation into slight retroversion while subsiding.

The two stem types showed equal potential in restoring anteversion- and GO symmetry within the range of ± 2.5° and ± 2.5 mm between sides. Further, there was no indication that neither anteversion- nor GO symmetry influenced postoperative migration. It therefore seems to be of no importance whether we choose a modular or a standard stem with regard to postoperative stem migration.

The stem of the ABG system is designed for a close anatomical proximal fit in the femur, which makes the stem version difficult to direct without modular options. Further, the standard stem has an offset that increases with size but limits the possibility for achieving a predetermined stem orientation. Stryker recalled the modular version of the ABG II system in June 2012 due to the potential for fretting and corrosion at the stem-neck junction [19]. A monolithic (standard) system with different offset and anteversion choices can compensate for the increased capabilities of a modular system to provide surgeons with options regarding anatomical restoration. With these increased options, we believe that a reliable preoperative template plan can give sufficient precision and accuracy in stem positioning regardless of what stem you use. We did not have preoperative access to the CT-based 3D templating software (Ortoma Plan^TM)^ which we later used to measure our anatomical parameters. Preoperative CT measurements done by a radiologist functioned as a guide for the surgeons during 2D templating and surgery. An asset to this study was that the observer, orthopedic surgeon, which made the radiological measurements for this study based on Ortoma Plan^TM^ was not involved in patients’ clinical follow-up and did not take part in their management. 3D templating software is superior to 2D templating because it gives information on hip version, and likewise, the conception of true femoral offset can be improperly assessed during 2D templating as well [20].

Although 3D-CT makes it possible to measure the leg length difference taking into account points in the hip, knee, and ankle for various positions of the legs and any valgus/varus deformities, we did not include the ankle in our CT analysis, and therefore, we could regrettably not include LLD in our study. There have been concerns regarding choosing appropriate and reproducible anatomical landmarks for 3D-CT measurements of anteversion caused by variability in dimension and contours of anatomical structures [21, 22]. This is particularly true for the trochanteric area proximal to the trochanter minor. We, therefore, decided to put the proximal reference point at the lower level of trochanter minor. The center is easily reproduced at this level whereas the medullary canal becomes more circular. We believe this better represents the longitudinal axis of the stem.

In the design of this study, we overestimated the effect anatomical parameters would have on the stem movement. The study design was underpowered for detecting the minor effect that anatomical parameters possibly have on postoperative migration of uncemented stems.

With the purpose of achieving better symmetry, it could be argued that a limitation of this study is the lack of divergence in anatomical restoration. This and the good stability of the stem used makes it hard to find any clinically important differences regarding stem migration. Based on our data, we cannot conclude to what degree we must restore symmetry to gain adequate stability for prosthetic parts. We will continue to evaluate the functional benefit of anatomical restoration by analyzing our study subjects further with data obtained from 3D gait analysis and correlate with different factors of anatomical reconstruction.

## Conclusions

Our results show a generally good symmetrical anatomical restoration and a benign migratory behavior with early stabilization for both types of the ABG II stem. Modular stems may allow better precision in GO reconstruction.

## Data Availability

The principal investigator, Dr. S. Kiernan, had full access to all of the data in the study and takes responsibility for the integrity of the data and the accuracy of the data analysis.

## Abbreviations

AO: Acetabular offset
CAS: Computer-assisted surgery
EGS: Elementary geometry shape
FO: Femoral offset
FO/AO: Femoral offset/acetabular offset quota
GO: Global offset
LLD: Leg-length-discrepancy
OA: Osteoarthritis
RSA: Radiostereometrical analysis
THR: Total hip replacements

## References

[1] Cassidy KA, Noticewala MS, Macaulay W, et al. Effect of femoral offset on pain and function after total hip arthroplasty. *J Arthroplast* 2012; 27: 1863-1869. 2012/07/20. DOI: S0883-5403(12)00310-5; 10.1016/j.arth.2012.05.001 [doi]. 10.1016/j.arth.2012.05.001 22810007

[2] Iversen MD, Chudasama N, Losina E, et al. Influence of self-reported limb length discrepancy on function and satisfaction 6 years after total hip replacement. *J Geriatr Phys Ther* 2011; 34: 148-152. 2011/09/23. DOI: 10.1519/JPT.0b013e31820e16dc [doi] 00139143-201107000-00007. 10.1519/JPT.0b013e31820e16dc PMC3179609 21937905

[3] Rosler J and Perka C. The effect of anatomical positional relationships on kinetic parameters after total hip replacement. *Int Orthop* 2000; 24: 23-27. 2000/04/25. 10.1007/s002640050006 PMC3619855 10774857

[4] Terrier A, Levrero Florencio F and Rudiger HA. Benefit of cup medialization in total hip arthroplasty is associated with femoral anatomy. *Clin Orthop Relat Res* 2014; 472: 3159-3165. 2014/07/18. DOI: 10.1007/s11999-014-3787-3 [doi]. 10.1007/s11999-014-3787-3 PMC4160515 25030101

[5] Little NJ, Busch CA, Gallagher JA, et al. Acetabular polyethylene wear and acetabular inclination and femoral offset. *Clin Orthop Relat Res* 2009; 467: 2895-2900. 2009/05/05. DOI: 10.1007/s11999-009-0845-3 [doi]. 10.1007/s11999-009-0845-3 PMC2758973 19412648

[6] Charles MN, Bourne RB, Davey JR, et al. Soft-tissue balancing of the hip: the role of femoral offset restoration. *Instr Course Lect* 2005; 54: 131-141. 2005/06/14. 15948440

[7] Kiernan S, Hermann KL, Wagner P, et al. The importance of adequate stem anteversion for rotational stability in cemented total hip replacement: a radiostereometric study with ten-year follow-up. *Bone Joint J* 2013; 95-B: 23-30. 2013/01/12. DOI: 95-B/1/23; 10.1302/0301-620X.95B1.30055 [doi]. 10.1302/0301-620X.95B1.30055 23307669

[8] Wretenberg P, Hugo A and Brostrom E. Hip joint load in relation to leg length discrepancy. *Med Devices (Auckl)* 2008; 1: 13-18. 2008/07/01. 10.2147/mder.s3714 PMC3417904 22915902

[9] Maloney WJ and Keeney JA. Leg length discrepancy after total hip arthroplasty. *J Arthroplasty* 2004; 19: 108-110. 2004/06/11. DOI: S0883540304001366. 10.1016/j.arth.2004.02.018 15190563

[10] Scheerlinck T. Primary hip arthroplasty templating on standard radiographs. A stepwise approach. *Acta Orthop Belg* 2010; 76: 432-442. 2010/10/27. 20973347

[11] Hassani H, Cherix S, Ek ET, et al. Comparisons of preoperative three-dimensional planning and surgical reconstruction in primary cementless total hip arthroplasty. *J Arthroplast* 2014; 29: 1273-1277. 2014/02/08. DOI: S0883-5403(14)00005-9; 10.1016/j.arth.2013.12.033 [doi]. 10.1016/j.arth.2013.12.033 24502952

[12] Mainard D, Barbier O, Knafo Y, et al. Accuracy and reproducibility of preoperative three-dimensional planning for total hip arthroplasty using biplanar low-dose radiographs : A pilot study. *Orthop Traumatol Surg Res* 2017; 103: 531-536. 2017/03/23. DOI: S1877-0568(17)30068-3; 10.1016/j.otsr.2017.03.001 [doi]. 10.1016/j.otsr.2017.03.001 28323248

[13] El Bitar YF, Jackson TJ, Lindner D, et al. Predictive value of robotic-assisted total hip arthroplasty. *Orthopedics* 2015; 38: e31-e37. 2015/01/23. DOI: 10.3928/01477447-20150105-57 [doi]. 10.3928/01477447-20150105-57 25611417

[14] Sakai T, Sugano N, Ohzono K, et al. Femoral anteversion, femoral offset, and abductor lever arm after total hip arthroplasty using a modular femoral neck system. *J Orthop Sci* 2002; 7: 62-67. 2002/01/31. DOI: 10.1007/s007760200010 [doi]. 10.1007/s776-002-8418-7 11819134

[15] Muller M, Abdel MP, Wassilew GI, et al. Do post-operative changes of neck-shaft angle and femoral component anteversion have an effect on clinical outcome following uncemented total hip arthroplasty? *Bone Joint J* 2015; 97-B: 1615-1622. 2015/12/08. DOI: 97-B/12/1615; 10.1302/0301-620X.97B12.34654 [doi]. 10.1302/0301-620X.97B12.34654 26637674

[16] Dorr LD, Faugere MC, Mackel AM, et al. Structural and cellular assessment of bone quality of proximal femur. *Bone* 1993; 14: 231-242. 1993/05/01. DOI: 8756-3282(93)90146-2. 10.1016/8756-3282(93)90146-2 8363862

[17] Geijer M, Rundgren G, Weber L, et al. Effective dose in low-dose CT compared with radiography for templating of total hip arthroplasty. *Acta Radiol* 2017; 58: 1276-1282. 2017/03/30. DOI: 10.1177/0284185117693462 [doi]. 10.1177/0284185117693462 28347158

[18] Valstar ER, Gill R, Ryd L, et al. Guidelines for standardization of radiostereometry (RSA) of implants. *Acta Orthop* 2005; 76: 563-572. 2005/10/01. DOI: V42136W7L1733G68; 10.1080/17453670510041574 [doi]. 10.1080/17453670510041574 16195075

[19] Molloy DO, Munir S, Jack CM, et al. Fretting and corrosion in modular-neck total hip arthroplasty femoral stems. *J Bone Joint Surg Am* 2014; 96: 488-493. 2014/03/22. DOI: 1840111; 10.2106/JBJS.L.01625 [doi]. 10.2106/JBJS.L.01625 24647505

[20] Weber M, Woerner ML, Springorum HR, et al. Plain radiographs fail to reflect femoral offset in total hip arthroplasty. *J Arthroplast* 2014; 29: 1661-1665. 2014/05/27. DOI: S0883-5403(14)00195-8; 10.1016/j.arth.2014.03.023 [doi]. 10.1016/j.arth.2014.03.023 24857334

[21] Hermann KL and Egund N. CT measurement of anteversion in the femoral neck. The influence of femur positioning. *Acta Radiol* 1997; 38: 527-532. 1997/07/01. 10.1080/02841859709174381 9240672

[22] Murphy SB, Simon SR, Kijewski PK, et al. Femoral anteversion. *J Bone Joint Surg Am* 1987; 69: 1169-1176. 1987/10/01. 3667647

